# Editorial: The role of bioactive compounds and nutrients in intestinal mucosal immunity, liver and vascular inflammation

**DOI:** 10.3389/fimmu.2025.1762284

**Published:** 2025-12-16

**Authors:** Fengjie Sun, Zheng Ruan, Tarek Gamal Abedelmaksoud

**Affiliations:** 1 Department of Biological Sciences, School of Science and Technology, Georgia Gwinnett College, Lawrenceville, GA, United States; 2 School of Food Science, Institute of Nutritional Sciences, Nanchang University, Nanchang, China; 3 Department of Food Science, Faculty of Agriculture, Cairo University, Oula, Egypt

**Keywords:** bioactive compound, gut microbiota, intestinal mucosal immunity, liver, nutrient, vaccine adjuvant, vascular calcification, vascular inflammation

As a major component of the human immune system, the intestinal mucosal immune system plays a critical role in preventing a wide range of gastrointestinal diseases (1–4). Intestinal inflammation is a key pathogenic factor in disorders including *Helicobacter pylori*-induced gastritis, eosinophilic gastroenteritis, inflammatory bowel disease (IBD), pancreatitis, and autoimmune hepatitis (5–10). Meanwhile, immune imbalances and various liver diseases are associated with the disruptions in intestinal homeostasis (11, 12). As a critical frontline immune organ, the liver processes gut-derived signals, including bacterial products and dietary antigens, to maintain immune-tolerance homeostasis (13–15). To date, the prevalence and incidence of non-alcoholic fatty liver disease (NAFLD) and alcoholic liver disease (ALD) continue to rise, contributing to an increased burden of cirrhosis and liver cancer (16, 17). Additionally, the incidence of autoimmune liver diseases (AILDs) is rising, although it remains relatively low in developing countries (18–20). Liver diseases remain among the top ten global causes of death.

To date, it is well known that bioactive compounds and nutrients play a key role in improving NAFLD, cardiovascular disease, obesity, IBD, and other diseases by regulating immunity (21–25). Due to their safety and effectiveness, bioactive compounds and nutrient interventions are widely considered beneficial for treating these diseases (26, 27). Therefore, it is important to summarize the recent advancements in the associations among bioactive compounds, nutrients, gut microbiota, and human health, demonstrating the significance of these interconnected factors in maintaining physiological balance and preventing disease, shaping therapeutic strategies, elucidating the underlying mechanisms that drive health and disease, and developing targeted nutritional and biomedical interventions.

Our Research Topic aimed to examine recent advancements, focusing mainly on the following three areas: (1) immunomodulatory effects and mechanisms of bioactive compounds and nutrients on intestinal immunity; (2) immunomodulatory effects and mechanisms of bioactive compounds and nutrients in NAFLD; and (3) metabolic characteristics and immune regulatory mechanisms of bioactive compounds and nutrients.

A total of four publications contributed to this Research Topic, including one review and three original research articles. In their review, Ma et al. systematically summarized the molecular mechanisms by which gut microbiota-derived bioactive compounds, i.e., short-chain fatty acids (SCFAs), bile acids (Bas), tryptophan derivatives, trimethylamine N-oxide (TMAO), endotoxins, and bacterial extracellular vesicles, regulated the development of MAFLD, explored the feasibility of using specific gut microbial metabolite profiles for MAFLD diagnosis, and highlighted potential therapeutic strategies targeting microbiota-host metabolic interactions. These strategies included the use of engineered bacteria to produce specific metabolites, probiotic/prebiotic interventions, and the clinical prospects of fecal microbiota transplantation. The authors indicated that the gut microbiota-derived bioactive compounds directly or indirectly modulated hepatic lipid metabolism, gut barrier integrity, immune responses, and signaling pathways, e.g., farnesoid X receptor (FXR), Toll-like receptor 4 (TLR4), and denosine monophosphate–activated protein kinase (AMPK). In clinical settings, distinct microbial metabolite profiles offer new diagnostic biomarkers, including propionate/butyrate ratios for SCFAs and 12-OH/non-12-OH BA ratio, thereby enhancing diagnostic precision. From a therapeutic perspective, interventions targeting gut microbiota-host metabolic interactions, such as high-fiber diets, probiotics/prebiotics, fecal microbiota transplantation, engineered bacteria, and postbiotics, show considerable promise for the treatment of MAFLD. In addition, the authors identified many areas that warrant further exploration. At present, defining the role of gut microbiota-derived bioactive substances in MAFLD continues to pose significant challenges. First, despite evidence supporting the benefits of SCFA supplementation, there are also reports of minimal effectiveness or potential adverse outcomes. Similarly, inconsistent results regarding BAs and TMAO underscores the necessity for stricter control of experimental variables to delineate their beneficial and adverse ranges, thereby facilitating their clinical translation. Second, individual microbiome heterogeneity reduces diagnostic consistency.

Zhu et al. constructed a dextran sulfate sodium (DSS)-induced mouse model of ulcerative colitis (UC), a chronic form of IBD characterized by recurrent mucosal inflammation that results in symptoms such as bloody diarrhea and weight loss, severely compromising patients’ quality of life. The aim of this study was to investigate the protective effects and underlying mechanisms of 2-(Indol-3-ylmethyl)-3,3′-diindolylmethane (LTr1), a trimeric compound derived from indole-3-carbinol (I3C), a natural bioactive molecule abundant in cruciferous vegetables and well recognized for its anticancer effects (28). The authors assessed the clinical symptoms, histological changes, and pro-inflammatory cytokine levels in the colon and serum of the mice as well as the macrophage infiltration and polarization in the colon and spleen. In addition, network pharmacology was used to reveal the molecular mechanisms underlying the therapeutic effects of LTr1. The main discoveries were as follows: LTr1 markedly improved clinical symptoms, mitigated histological injury, maintained intestinal barrier integrity, and reduced inflammatory cytokine production; LTr1 also inhibited DSS-induced macrophage infiltration and M1 polarization *in vivo*, and directly suppressed lipopolysaccharide-induced M1 macrophage polarization *in vitro*. Furthermore, network pharmacology analysis revealed multiple proteins and signaling pathways (i.e., TP53, AKT1, HSP90AA1, EGFR, and SRC) as potential mediators of LTr1’s effects in UC. This study establishes a theoretical basis for improving UC treatment strategies and underscores LTr1 as a promising candidate for new therapeutic development.

Due to the limitations of the Shingrix^®^ vaccine adjuvant, Wang et al. developed a novel adjuvant system, BK-02, which comprised the TLR9 agonist BK-02C (CpG2006) and a squalene-based oil-in-water emulsion, BK-02M (MF59). When paired with glycoprotein E (gE), the active ingredient of the recombinant herpes zoster (HZ) vaccine, the BK-02 adjuvant system induced markedly higher gE-specific IFN-γ^+^ T-cell responses (486 SFU/10^6^ cells, a 121-fold increase compared with gE alone) and elevated IgG antibody titers (Lg titers 5.2 vs. 3.4 for gE alone). The authors determined that the optimal dose was 5 μg gE + 30 μg BK-02C + 1× BK-02M, which corresponded to a clinical dose of 50 μg gE + 300/500 μg BK-02C + 0.5 mL BK-02M. Additionally, pilot-scale samples of the recombinant HZ vaccine elicited stronger gE-specific CD4^+^ and CD8^+^ T-cell responses compared with Shingrix^®^. Furthermore, the gE/BK-02 adjuvant system induced a Th1-regulated mixed immune response, promoting strong cellular and humoral immunity. This study provides a promising adjuvant candidate for the current HZ subunit vaccines. The authors identified the following limitations and proposed potential solutions. Since HZ primarily affects elderly populations with weakened cell-mediated immunity (29, 30), future experiments should include aged mouse models. Moreover, because varicella zoster virus (VZV) is highly species-specific and causes clinical symptoms only in humans and non-human primates, murine models cannot support viral latency or reactivation (31). Thus, thorough efficacy assessment in rhesus macaque models is a crucial step in preclinical development. In addition, future studies should incorporate neutralization assays to more comprehensively evaluate the protective capacity of antibodies elicited by the BK-02 adjuvant system.

Zhang et al. investigated the anti-calcification effects of epigallocatechin-3-gallate (EGCG), a type of natural polyphenol, in relation to gut microbiota and metabolite remodeling in mice. They first established a vitamin D3-induced calcification model of mice and then treated the mice with EGCG for 11 weeks. The primary findings were presented in four areas. (1) EGCG significantly inhibited calcification (*P* < 0.05), as shown by decreased alizarin red S staining and reduced alkaline phosphatase activity. (2) EGCG restored alpha diversity and induced taxonomic shifts in gut microbiota at various taxonomic levels. (3) Serum metabolomics revealed that EGCG reversed the vitamin D3-induced upregulation of phospholipid metabolites. (4) EGCG promoted ubiquinone biosynthesis and activated terpenoid-quinone pathways. This study establishes EGCG as a dual modulator of the gut microbiota and host metabolites, with therapeutic potential against vascular calcification, providing a novel approach to target gut-vascular crosstalk in cardiovascular disease.

In summary, these contributions collected in this Research Topic underscore recent progress in the relevant areas of human health and identify future research directions for further exploration. Given the innovative technologies for investigating the interactions among bioactive compounds, nutrients, and human health, substantial advancements in human health are highly expected based on the areas covered in this Research Topic.
